# Recent Advances in Vision-Based On-Road Behaviors Understanding: A Critical Survey

**DOI:** 10.3390/s22072654

**Published:** 2022-03-30

**Authors:** Rim Trabelsi, Redouane Khemmar, Benoit Decoux, Jean-Yves Ertaud, Rémi Butteau

**Affiliations:** 1 Normandie University, UNIROUEN, ESIGELEC, IRSEEM, 76000 Rouen, France; rim.trabelsi@ymail.com (R.T.); benoit.decoux@esigelec.fr (B.D.); jean-yves.ertaud@esigelec.fr (J.-Y.E.); 2 Normandie University, UNIROUEN, UNILEHAVRE, INSA Rouen, LITIS, 76000 Rouen, France; remi.boutteau@univ-rouen.fr

**Keywords:** holistic on-road behavior analysis, driver-road interaction, driving status analysis, road scene understanding, situational awareness, trajectories forecast, deep learning, review

## Abstract

On-road behavior analysis is a crucial and challenging problem in the autonomous driving vision-based area. Several endeavors have been proposed to deal with different related tasks and it has gained wide attention recently. Much of the excitement about on-road behavior understanding has been the labor of advancement witnessed in the fields of computer vision, machine, and deep learning. Remarkable achievements have been made in the Road Behavior Understanding area over the last years. This paper reviews 100+ papers of on-road behavior analysis related work in the light of the milestones achieved, spanning over the last 2 decades. This review paper provides the first attempt to draw smart mobility researchers’ attention to the road behavior understanding field and its potential impact on road safety to the whole road agents such as: drivers, pedestrians, stuffs, etc. To push for an holistic understanding, we investigate the complementary relationships between different elementary tasks that we define as the main components of road behavior understanding to achieve a comprehensive understanding of approaches and techniques. For this, five related topics have been covered in this review, including situational awareness, driver-road interaction, road scene understanding, trajectories forecast, driving activities, and status analysis. This paper also reviews the contribution of deep learning approaches and makes an in-depth analysis of recent benchmarks as well, with a specific taxonomy that can help stakeholders in selecting their best-fit architecture. We also finally provide a comprehensive discussion leading us to identify novel research directions some of which have been implemented and validated in our current smart mobility research work. This paper presents the first survey of road behavior understanding-related work without overlap with existing reviews.

## 1. Introduction

Vision-based Advanced Driver-Assistance Systems (ADAS) and Autonomous Vehicles (AV) development has been witnessing a slow yet impressive growth over the past two decades for most of its modules including sensing hardware [1], perception [2], mapping and path planning [3], and scene understanding [4]. Despite the large amount of research and development endeavors, the area still lacks comprehensive approaches and/or systems which provide high-level road scene analysis that includes holistic description of the AV’surrounding. By contrast to most of the ADAS and AV vision-based tasks, there are scarce resources for broad road scene understanding, which is mainly due to the paucity of benchmarks, standards, theoretical fundamentals, and expressly research papers that urge the community to tackle this task.

**Figure 1:**
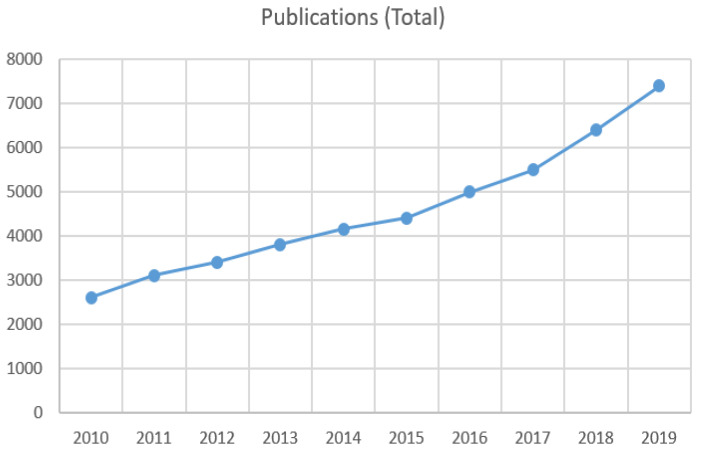
The increasing number of publications in road scene understanding from 2010 to 2019. Data from Google Scholar advanced search: allintitle: “on-road behavior understanding” AND “on-road behavior analysis ”.

To push forward this research area, we need to outline the milestones and the recent tools and approaches to the community so that we allow for an easier recognition of the required gait. For this purpose, we need to expose not only the required steps but also a tutorial that highlights the perks and the limitations of each method, which will allow researchers to choose the appropriate solution for their context, which inherently includes trade-offs. In this survey paper, we are going to take all these aspects in order to review the most successful papers that have been published in recent years. Thanks to the onset and the impressive development of the revolutionary deep learning approaches [5,6], new blood has been brought into on-Road Behavior Understanding (RBA) tasks, creating notable improvements and drawing attention to new perspectives. Figure 1 illustrates the increasing number of publications related to “on-road behavior understanding” since 2010.

The key contributions of this original review paper can be summarized as follows.

1. We present the first-ever background information on our foreground topic, RBA, and do this by providing the reader with all the required information, theoretical concepts, and keywords to read the remainder of the survey and boost their attention to the approaches we are going to point out;
2. We link fragmented tasks related to vision-based RBA in a coherent context so that the reader can understand the complementary relationships between all the elementary aspects and makes use of them in order to achieve a holistic RBA;
3. We identify common concepts and features adopted by the milestone and the recent approaches so that we can draw fair comparisons and critics;
4. We stress the contribution of deep neural networks approaches by categorizing all the reviewed papers depending on the kind of the meta-architecture used;
5. We present a comprehensive comparison for all the recent publicly available datasets and we list all the metrics used in the state-of-the-art to assess the performance of RBA-related tasks;
6. Critical analysis of the overall topic is finally elaborated, leading us to draw consistent future directions.

**Figure 2:**
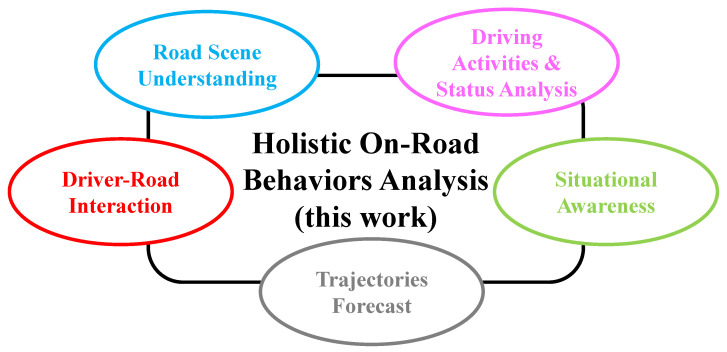
A pictorial description of the contents of this work, i.e., on-road behavior analysis components: situational awareness, driver-road interaction, driving activities and status analysis, trajectories forecast, and road scene understanding along with holistic endeavors that provide a broad understanding of road environments.

The remainder of this paper is organized as follows. In Section 2, we review all the closely related surveys. Then, in Section 3, we emphasize the gravity and challenges of such high-level analysis of visual content for ADAS and AV systems. Following the classification of elementary aspects illustrated in Figure 2, the state-of-the-art approaches are summarized in Section 4. Deep (meta)-architectures and the feature extractors they encapsulate are then presented in Section 5. Section 6 exposes a comprehensive study of the available benchmarks. We conclude our paper in Section 7 by providing critiques and challenges of the overall literature review and future directions.

## 2. Related Reviews

A number of surveys of general vision-based ADAS and AV approaches have been proposed recently [7,8,9,10]. Their major contributions and the differences with our work are presented in what follows.

**Deep reinforcement learning for general AV tasks.** An original yet scanty analysis of deep reinforcement learning for AV systems is conducted in this review [7]. The employ of deep reinforcement learning is becoming crucial in such real-world applications. This paper promotes the use of reinforcement learning for AV vision-based tasks by providing the fundamentals of state-of-the-art algorithms and addressing the issues to overcome in order to succeed in its reuse with AV systems. This issue has never been covered by previous surveys, but the authors did not review papers related to a broad descriptions of the road environments. They explicitly presented unrelated works relevant to diverse applications such as vehicle control [11], vehicle overtaking [12], and ramp merging[13] among others. In our work, we also include all the deep learning approaches while providing links between all the components of on-road behavior analysis.

**Comprehensive review for detection, tracking, and behavior analysis.** The survey proposed in [9] extensively reviews papers dealing with three topics, i.e., detection, tracking, and behavior analyzing for AV systems, spanning over eight years between 2005 and 2013. This survey helps build a broad knowledge of the semantic interpretation of road scenes. The approaches reviewed are all based on handcrafted and classic machine learning algorithms. The review process follows a categorization in regard to sensing systems, mainly, monocular vision, stereovision, and their combination. Even though the authors claim that they are proposing a novel survey for vehicle behavior as the emerging task of AV systems, they fail to motivate the community of (i) its importance and (ii) the required knowledge and the way to formalize such types of high-level problems, which will be the object of our work.

**Secure intelligent systems based on deep neural networks.** An in-depth investigation of the success behind deep learning technologies on such intelligent systems is conducted in [10]. Through the examination of around 223 works, the main objective of their proposal is to explain for the community how the decisions of deep neural network black-boxes are made (we refer the reader to these two surveys [14,15] for further details regarding explainable artificial intelligence fundamentals). Bringing the attention of the community to such an issue is of great importance for the security and the safety of AV systems through the standardization of quality assurance rules and systematic engineering guidance. Despite the novelty of the addressed issues, their paper lacks fundamental analysis related to AV and ADAS systems, among others, and remains a very general overview of the above concerns.

**Figure 3:**
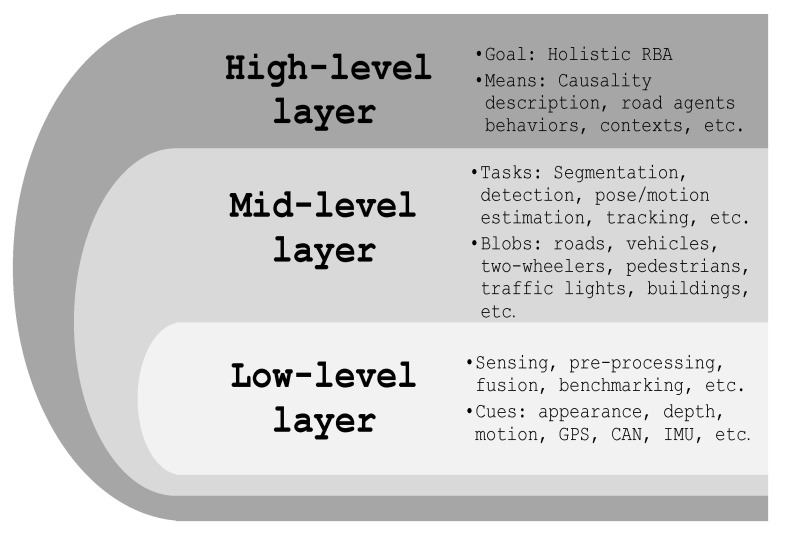
Illustrating the ascending levels of on-road behavior analysis. At the lowest layer, data such as appearance, depth, and motion are collected, pre-processed, and labeled to prepare the settings for higher layers’ tasks. One level up, several mid-level understanding scenarios might occur to describe and model spatial and/or temporal visual data like road agents detection, recognition, activities classification, maneuvers identifications, etc. At the highest level, an aggregate of the lower layers’ outcomes is used to learn the overall on-road behaviors by including more intuitive paradigms like causality. In this work, we focus on five high-level tasks, mainly situational awareness, driver-road interaction, road scene understanding, trajectories forecast, and driving status analysis.

In Ref. [7], the understanding of a scene provides high-level scene comprehension (e.g. Figure 3) that includes detection, classification, and localization tasks, feeding the driving policy/planning module. Path Planning predicts the future actor trajectories and maneuvers. A static shortest path from point A to point B with dynamic traffic information constraints is employed to calculate the path.

## 3. Importance and Challenges of On-Road Behaviors Analysis

Road crashes kill nearly *“1.25 million people each year, on average 3287 deaths a day and around 50 million are injured or disabled”,* reports the Association for Safe International Road Travel (ASIRT) (https://www.asirt.org/, accessed on 1 December 2021). By 2030, fatal traffic crashes are expected to become the fifth leading cause of death worldwide. ITS (Intelligent Transportation Systems) is investigating to find smarter solutions that allow bolder ADAS and AV systems in order to reduce road fatalities.

To do so, many vision-based components have been developed and significant advances have been made on semantic segmentation [16], object detection [17], mapping and localization [3], etc. Yet, such tasks have not really addressed the challenges in high-level understanding and correspond only to the first step of understanding, thus the importance of on-road scene analysis. In fact, detecting/localizing participants in a given scene and parsing them to the semantic classes is just a mid-level analysis step and we believe that to avoid road crashes it is crucial to understand the context and the interactions between the stuffs of the road and the participants’ behaviors and their status.

**Figure 4:**
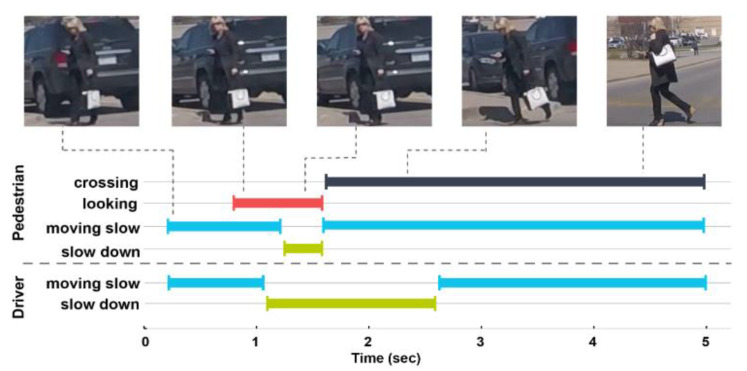
Example of pedestrian and driver behaviors represented over time for an event that took place while crossing and their corresponding causality [18].

**Figure 5:**
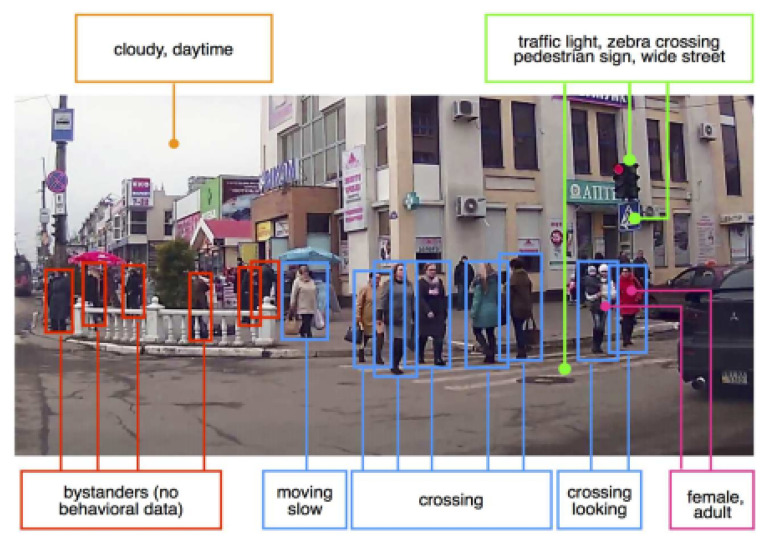
Cluttered road scene with extensive annotation setting that includes bounding boxes for all events, behaviors, and objects information along with the contextual labels [18].

Despite the ITS community always asking “what are the difficulties and challenges in on-road behaviors analysis?”, this question has not been raised seriously so far in the literature or even been generalized. As vision-based understanding related tasks have different goals and constraints, integrating a set of these tasks under the same framework implies imperatively a higher complexity. Aside from common issues in image and video analysis tasks such as sudden and gradual illumination changes and viewpoints variation, the specificity of road environments involves more aspects including temporal and spatial patterns intra-class variations (cf. Figure 4), cluttered scenes (cf. Figure 5), and the curse of data labeling for high-level analysis.

## 4. RBA Components and Milestones over the Last 20 Years

In the last two decades, it has been widely accepted that the progress of road scenes analysis has generally gone through two historical periods: (i) traditional understanding methods era (before 2013) and (ii) deep learning-based understanding era (after 2013). In this section, we are going to highlight the state-of-the-art methods that have been proposed over the last 20 years for each of the RBA’s components we identify from the literature, mainly situational awareness, driver-road interaction, road scene understanding, trajectories forecast, and driving activities and status analysis.

### 4.1. Situational Awareness

Situational awareness is a fundamental ingredient for successful on-road scene analysis. In fact, while dealing with the perception of road environments, including objects, participants, behaviors, and activities, we need to take into consideration their meaning, and their future status in conjunction with space and time.

For example, Kooij et al. propose in [19] a pedestrian situational awareness aiming to predict a pedestrian’s path in the AV domain. To anticipate the decision of pedestrians, they assume that it is influenced by three factors: (i) the presence of an approaching vehicle on a collision course, (ii) the pedestrian’s safety awareness, and (iii) the specificity of the environment. To incorporate these factors, latent states have been built on switching linear dynamical systems and dynamic Bayesian networks to anticipate changes in pedestrian paths. The situational awareness evaluates whether the pedestrian at a given instant *t* has seen the vehicle previously (before *t*)) through the estimation of the distance between vehicle and pedestrian at the expected point of closest approach head orientation and his distance to the road curbside. The ultimate goal of this work is to predict the intention of a pedestrian to laterally cross the road, which is highly related to most of the pedestrian fatalities inroads according to accident analysis reported in [20]. For a similar objective, in [18] the crossing behavior, i.e., the pedestrians’ intention to cross, is predicted. The authors consider here two classification tasks of static environmental context and passenger action. Due to the lack of annotation of the JAAD dataset proposed in this same paper, they make use of weakly supervised learning with CNN (Convolutional Neural Network) features through an AlexNet architecture to identify visual attributes for both tasks [21]. The considered classes for the environmental contextual recognition task include the following elements: narrow, wide, pedestrian crossing sign, zebra crossing, stop sign, traffic light, and parking lot. As for the pedestrian action classification, only looking and crossing classes have been considered. Their experimental results prove that using only the pedestrian action information can predict 40% of the observed crossing behavior, yet, adding situational awareness information/context improves the results by 20%.

### 4.2. Driver-Road Interaction

Understanding the interactions occurring between drivers and road users is crucial toward a holistic RBA. Recently, the ITS community has brought attention to this issue by developing new vision-based models that capture the interaction between road users and the ego vehicle.

Owing to the newly published HDD dataset [22], Li et al. propose in [23] a spatio-temporal 3D-aware framework by means of GCN (Graph Convolution Networks). For this purpose, participants and objects of the road scene are categorized into two sets: Thing objects, such as pedestrians and cars, and Stuff objects, such as traffic lights and road surface marking. As an example, interactions with Thing objects may include stopping to let pedestrians cross and deviation for parked vehicles, and examples for interactions with Stuff objects may include changing or merging a lane. To represent the interaction between these sets of objects and the ego vehicle, two kinds of graphs have been proposed: Ego-Thing Graph and Ego-Stuff Graph to represent the ego vehicle interaction with Thing and Stuff objects, respectively by extending the Ego-Thing Graph proposed in [24]. In addition, two contributions have been introduced: the incorporation of the 3D location of objects and the definition of an Ego Node as the ego vehicle. Thus the proposed approach offers higher performance in terms of mAP (mean Average Precision) and outperforms other baselines on HDD dataset by 6% and 3.9% for Thing and Stuff sets, respectively. Among methods that have been surpassed by the above approach, we can list the work proposed in Ref. [22] as baselines for the HDD dataset. Their proposal encodes implicitly the states of the ego vehicle and all the other objects using usual CNN features and RNN (Recurrent Neural Network) units.

Previously, in 2000, using traditional machine learning tools in Ref.[25], HMM (Hidden Markov Model) was leveraged to recognize the tactical driver behavior. To model the states of the ego vehicle and all the elements and participants in the road environment, a single node was used for each one and then all the nodes were encoded into a state vector. This approach is not reporting state-of-the-art results so far, but it still inspires recent endeavors like those stated earlier.

### 4.3. Road Scene Understanding

ITS and computer vision communities have exerted extensive effort to analyze the road scene [26,27,28]. As hinted to earlier, traditional approaches were handcrafted or machine learning-based and they were designed to deal with an elementary task such as detection recognition or segmentation for a specific kind of object lane [29], traffic lights [30], and pedestrians [31]. Before 2013, it was not obvious how to get a holistic understanding of road environments. With the advent of the revolutionary deep neural networks, it was made possible to get a comprehensive understanding of the road scene. In fact, deep learning provides more automatic models, which allow combining many tasks in a single framework.

For example, Ref. [32] proposes an encoder-decoder architecture that provides an end-to-end road scene understanding. The encoder is a CNN-based network similar to VGGNet [21]. Similarly, the decoder is a CNN network with two streams that upsamples the features and then fuses the information of both streams to the decoder network, which outputs the score of each pixel with regards to the predefined classes. By reporting qualitative and quantitative evaluation done only over the CamVid dataset [33], the authors claim that this encoder-decoder network is able to provide higher results for semantic segmentation and positioning in terms of CA (Class Accuracy) at MIoU (Mean Intersection over Union). However, the use of VGGNet as a backbone also gives outcomes 68.49% and 56.05% for CA and MIoU, respectively, meaning that there is a lot to improve.

Under the same context, Ref. [16] deals with a more challenging task: recovery of occluded vehicles, in addition to the amodal segmentation task. A novel multi-task approach was introduced to incorporate the two tasks under the same framework. In another word, two networks were introduced, called (i) segmentation completion and (ii) appearance recovery. The first one aims to produce the recovered segmentation mask. Afterward, the obtained mask is used for the second network to produce the occluded parts of vehicles. As a result, the invisible regions are restored/painted back to the foreground of the original image. Experiments were done with the Occluded Vehicle Dataset (OVD) and metrics used to evaluate the recovered segmentation mask including recall, precision, F1 score, IuO, the per-pixel L1 error and the per-pixel L2 error were used. As for the recovered appearance of the vehicle task, the L1 and L2 errors have been used along with two new metrics: the ICP (Inception Conditional Probability) and the SS (Segmentation Score). Overall, the obtained results demonstrate similar performance to Deeplab [34] without the iterative refinement for the segmentation with a better capability of generating the invisible parts.

As for the work proposed in Ref. [35], the authors focus on urban scene analysis and road detection and evaluate their proposal over KITTI [36] and LabelMeFacade [37] datasets. The major contribution of this paper is the presentation of new convolutional patch networks learned for pixel-wise labeling in order to classify image patches. More details regarding the proposed CNN can be found in Section 5.

### 4.4. Trajectories Forecast

Trajectories forecast in road environments consists of predicting the path that a moving vehicle, pedestrian, or any other road agent follows through road space as a function of time. The extracted trajectories from a visual data are less mastered with relation to ADAS and AV contexts. In this survey, we highlight its importance and the methods developed by the computer vision community that can be useful in such circumstances. In addition to the prediction of future paths of road participant, this RBA component is of crucial importance and might be used to supply other components by modeling traffic representation in real-time. Thus, this task is useful for performing safe ADAS and AV systems not only by improving all the vision-based components but also by using it in the management of congestion and vehicle routing.

For instance, Ref. [38] proposes an approach named DROGON for “Deep RObust Goal-Oriented trajectory prediction Network” that forecasts future trajectories of vehicles by taking into consideration their behavioral intention, which is commonly called causal reasoning. A conditional prediction model has been built to solve this issue. Three steps have been followed to train such a model. The first step is to infer the interaction of vehicles with other road agents. Then, the second and the third steps consist in estimating their intention and thus by computing the intention’s probability distribution based on the inferred interaction and the causal reasoning, respectively. To evaluate their approach, Choi et al. also introduce a new dataset acquired at four-way intersections. Through qualitative and quantitative evaluation using ADE (Average Distance Error) and FDE (Final Distance Error) metrics, the DROGON framework has demonstrated an efficient performance for predicting and generating trajectories over the state-of-the-art.

A near-term trajectories forecast approach is also proposed by Chandra et al. in [39]. They tackle more challenging contexts, mainly heterogeneous traffic where road agents are very varied for, e.g., pedestrians, cars, bicycles, trucks, buses, motorcycles, etc. The major contribution of this paper is the representation of heterogeneous interactions between different kind of agents. An RNN-CNN framework called *TraPHic* is introduced in order to automatically predict the trajectories and does so by taking into consideration the challenges of heterogeneous contexts related to different textures, shapes, motions, and activities of each road participants. Several experiments have been carried out over the NGSIM dataset [40] and a new dataset called *TRAF* introduced in this paper prove that the proposed method outperforms the state-of-the-art by around 30%.

### 4.5. Driving Activities and Status Analysis

Driver’s status and activities analysis are crucial because one of the leading cause of traffic accidents is related to driver’s inattention, aggressive maneuvering, or drowsiness. Detecting or predicting strange maneuvers or status may help avoiding fatal crashes by keeping a distance to identified aggressive drivers.

**Figure 6:**
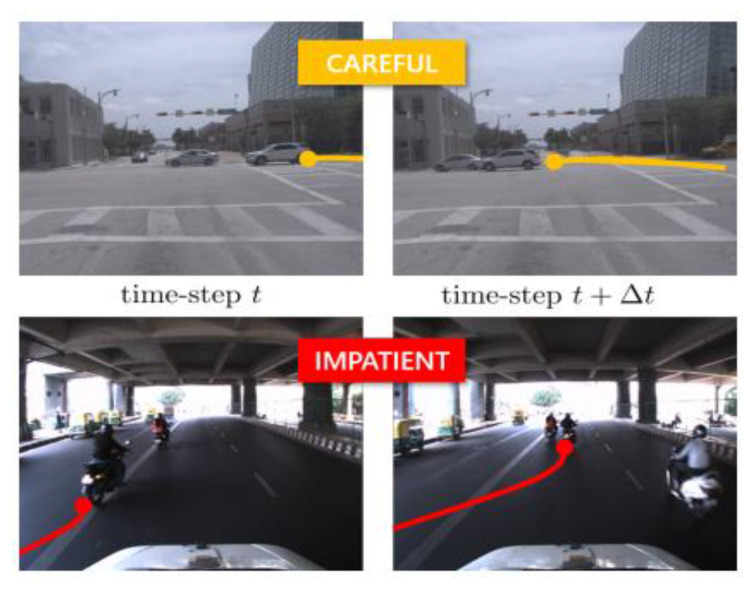
Trajectories generation produced in Ref. [41] for two different contexts. Through the traits shown by the road agent’s trajectory, their behaviors is then predicted.

In the literature, Ref. [41] proposes a novel framework named GraphRQI to learn, in a supervised fashion, third party driver behavior (Figure 6). Based on the approach proposed in Ref. [39] (detailed in the previous section), road participants trajectories are used to pinpoint the general traits of the driver, i.e., conservative or aggressive, which inherently affects the trajectories of the surroundings agents. By means of GCNs trained on the TRAF [39] and Argoverse [42] datasets, driving status has been classified into six classes: *reckless, careful, timid, impatient, threatening, and cautious*. The result in terms of accuracy is improved up to 15% and 25% on Argoverse and TRAF datasets, respectively.

In a similar way, Ref. [43] classifies the ego vehicle behavior through scene understanding-based CNN and robust temporal representation-based LSTM (Long Short Term Memory). The key element of this work is the incorporation of scene context features related to weather and road characteristic, places, types, and surface conditions. Experimental results shown on the HDD dataset and a newly collected dataset prove that the proposed scene classification boosts the understanding of driver behavior.

Multi-modality cues have been extensively used with regard to driver maneuvers classification for example, by taking into consideration only the temporal aspect, Ref. [44] presented a fancy architecture based on Gated Recurrent Fusion Unit to model multi-modal data coming from two streams: video and CAN bus. Novel gating functions have been introduced in order to represent the exposure of each data stream at each frame in order to provide an adaptive data fusion. To recognize the action that occurred during the driving scenarios over the HDD dataset, Ref. [45] employs a CNN-based architecture with a triplet loss as a space embedding regularizer to limit the problem of overfitting encountered with HDD pre-trained models. Extensive evaluation done on the HDD dataset proves that softmax plus triplet loss achieves a better performance on minority classes, proving a better generalization of the trained network.

Following a multi-modal framework as well, Ref. [46] presents DBUS (Driving Behavior Understanding System), which makes use of image sequences synchronized with GPS/IMU signals. The perks of DBUS is the recognition of the driver’s attention and intention along with the maneuver. A unified model has been proposed to jointly analyze the attention and the intention of the driver activity. Even though the proposal is original, its efficiency was not evaluated over state-of-the-art benchmarks and it has only been tested on a non publicly available dataset.

Using the same concept of multi-modal and multi-level understanding, Ref. [22] recognizes driver behaviors through different layers including attention, cause, stimulus-driven action, and goal-oriented action. Contrary to Ref. [46], the proposed approaches based on standard CNN and LSTM have been evaluated over the HDD benchmarks devoted to learning the driver’s activities and causal reasoning. The same model has been reproduced in [47] where Chen et al. propose to classify driving behavior through new modes, color, and depth sequences coming from video and LiDAR sensors, respectively. The introduced dataset will be detailed in Section 6.

### 4.6. Holistic Understanding

Holistic understanding aims at jointly dealing with different complementary tasks within the same framework for RBA. In the literature, there was a lack of serious endeavors until 2015. Jain et al. proposed the first-ever framework that incorporates several levels of understanding to predict maneuvers of the ego vehicle [48]. Both inside and outside contexts (with regards to the vehicle) have been taken into consideration. For the inside context, authors consider the visual representation for the driver’s facial expression and motion. As for the outside context, scene perception has been investigated to anticipate a driver’s maneuver through multi-modal features such as image sequences, GPS, road maps, speed, and events. Auto-regressive HMM models have been proposed to generate the latent states of the driver [49]. Owing to the uniqueness of the approach, promising results have been obtained on a first-ever dataset called Brain4Cars including multi-modal inside and outside data of the vehicle.

Ref. [50] introduces an end-to-end learning approach of driving models. Based on visual features of the current and the previous vehicle states, the contribution of this paper is the proposition of a holistic approach that predicts the distribution driving behaviors along with semantic segmentation. An adaptive FCN-LSTM model has been proposed to anticipate the distribution of the vehicle motion (cf. Section 5). A crowd-sourced driving behavior dataset is also introduced to better evaluate the efficiency of the proposed learning paradigm for both semantic segmentation and behaviors prediction.

A more comprehensive approach was introduced in [51] to jointly analyze road agent dynamics, interactions, and the scene context along with the prediction of future states of the road agents, not only the ego vehicle as done by the previously cited work earlier in this section. A CNN architecture was used to encode static and dynamic states and semantic map information in a top-down spatial grid. Due to the lack of a benchmarking dataset that includes road map information, the authors propose a novel large-scale dataset to advance the state-of-the-art of the field (cf. Section 6 for further information).

**Figure 7:**
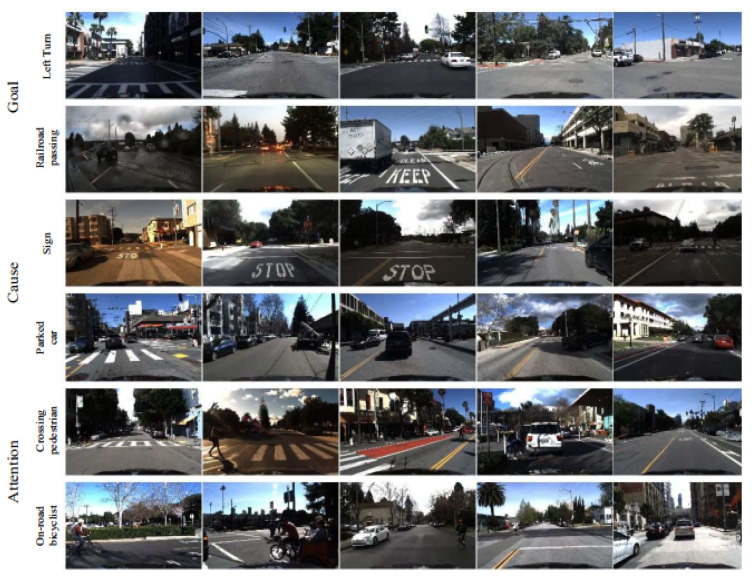
Divers on-road behavior scenarios acquired using an ego-vehicle. Four different annotation layers are proposed in the HDD dataset to describe the driver and the behaviors of his surroundings [22].

Peng et al. introduce in [17] the so-called ADMD (Attention-driven Driving Maneuver Detection) system, which investigates the causal relationship between the road scene and the driving maneuvers. Similar to Ref. [22], the driver’s attention is explored to boost the performance of their system (Figure 7). The major contribution of this work is the proposition of a distillation approach to transfer latent features into the maneuver detection system, which helps to discern a driver’s maneuvers from his attention areas. Experiments carried out on a new dataset prove that ADMD is able to achieve promising performances. This is basically owing to the novel learning paradigm capable to interpret the vehicle surroundings that might affect the intention of the driver’s maneuvers.

## 5. Deep Learning Solutions

The revolutionary deep learning has proven to be efficient in various fields including computer vision and image processing with application to various kinds of applications such as autonomous driving. In what follows in this section, we are going to report all the deep neural network architectures that have been used in the literature in the RBA tasks cited earlier in the previous section. Specifically, we are going to highlight the contribution of all the related state-of-the-art approaches based on the core architectures. The goal of this study is to let the practitioners decide the meta-architecture and feature extractors that are more suitable for their application.

### 5.1. Deep Convolutional Neural Networks

CNNs are the most appealing variant of deep neural networks in vision related-tasks. They were basically developed by LeCun et al. in [52] and successfully reused in the ILSVRC (ImageNet Large Scale Visual Recognition Challenge) competition by Krizhevsky et al. [21]. CNNs are able to perform feature learning to get consistent representation from visual data (basically). Its architecture includes three kinds of layers/mapping functions; convolution, pooling, and ended with one or more fully connected or GAP (Global Average Pooling) layers. Dropout and batch normalization is generally used in CNN for regularization. The biggest advantage is the use of weight sharing (contrary to conventional neural networks), which helps in saving memory and reducing complexity. Along with this, CNNs have shown impressive performance and outperformed all the machine learning techniques devoted to visual understanding.

One of the main application of CNNs is object detection, which represents an important task and a major challenge in computer vision [53]. Its challenges are mainly related to object localization and classification. Several methods based on CNN exist in the literature. Overall, methods using CNNs fall into two categories: (i) single-stage methods, which perform object localization and classification in a single network such as Single-Shot Detector (SSD) [54] and You Only Look Once (YOLO) [55], Both of these architectures produce as output a bounding box of each detected object, its class, and its confidence score [53], and (ii) two-stage methods, which have two separate networks for each of these tasks.

Among its milestones models, the important engines in CNN are AlexNet [21], VGG-16 [56], GoogleNet/Inception [57], ResNet [58], and Xception [59]. Autonomous driving systems take advantage of the so-called pre-trained model of an already-trained model over other visual tasks. Expressly, for a completely new problem/data, the ITS community makes use of the publicly available model to either (i) use open access meta-architectures to train it from scratch on their own data or (ii) use the pre-trained models as feature extractors by feeding new data to a trained model with its trained weights and tweak it to train the new task, which is what we call transfer learning. Commonly, the output layer is replaced with a new fine-tuned layer in order to determine the deviation of the prediction to the labeled data. Thus, using a non-large-scale dataset, three training ways might be considered: train only the output layer, train the whole CNN, or the last few layers. The ITS community makes use of all these learning strategies to build, train, or retrain their CNN backbones. With application to the on-road behavior tasks, while many endeavors succeed to generate robust features from pre-trained models [17,32,35], major improvements have been proposed recently at many levels of the standard architecture with regards to the spatial exploitation, depth, and feature map exploitation.

**Graph Convolutional Network (GCN).** GCN consists in using graph-based learning instead of learning data represented in the Euclidean space (such as image or video). The strength of the graph-based framework is the ease of representing the interaction occurring between the instance’s components. This is of great importance in road scenes, which will serve in describing the causality between the intention and the attention of the drivers and all the road participants. This paradigm has been exploited by Li et al. in [23] to model interactions, as detailed earlier in Section 4. The two proposed networks, Ego-Thing Graph and Ego-Stuff Graph advanced the state-of-the-art of GCNs through the extension of the backbone proposed in Ref. [60] with two-stream instead of one-stream. First, to generate graphs, 3D convolutions have been applied to obtain the first level of visual features which are feeding both streams; Thing and Stuff graphs. Then, as a second step, the Thing and Stuff representations are extracted using RoIAlign [61] and the newly proposed approach called MaskAlign to deal with irregular objects. The extracted features from both streams are then used to generate the graph generators using a frame-wise fashion. Ego-Thing Graph and Ego-Stuff Graph allow afterward to pass on the connections between different kinds of objects through these GCNs. Outputs from the two obtained streams are fused and fed into a temporal module. This latter module aggregates spatial features using max-pooling to compute the final GCN output.

**Fully Convolutional Networks (FCN).** Unlike standard CNN, FCN is an end-to-end network where a Fully Connected (FC) layer [62] or an MLP (Multi-Layer Perceptron) network [63] are derived on the top of a CNN-like network where filters are learned at all the levels including the decision-making layers. This allows FCNs to learn representations and scoring based on local features/data. Ref. [50] exploits this fact to propose an end-to-end architecture for generic motion models for autonomous vehicles under crowd contexts. The authors here propose the so-called dilated FCN approach. Taking advantage of the pre-trained CNN models, the second and the fifth pooling layers have been removed and a dilated convolution layer replaced the third convolution layer through FC7. The difference between the usual convolution with stride and the dilated one is the expansion of the filter’s size before doing the convolution.

Among other applications of FCN is semantic segmentation, where the output of the model has the same resolution as the input images, with a class prediction for each pixel. Since the pioneering work of Long et al. [64], where the base structure of the model is an encoder and a decoder streams, many variants have been proposed. Most of these works focus on the improvement of the accuracy of segmentation. However, real-time performance is very important for autonomous driving. Combination of light architectures like SkipNet [64] and ShuffleNet [65] for the encoder and decoder parts, respectively, allows segmentation rates at about 16 FPS on a Jetson TX2, while maintaining high accuracy [66].

**3D CNNs.** represent an extension of CNNs where a 3D activation map is generated over the convolution layers. The intuition behind this is to encode data that can be represented on more than two dimensions like volumetric or temporal data. 3D CNNs have been used in different contexts such as 3D shape estimation [67], human activity detection [68], and recently explored in RBA systems [69] to further enhance the understanding of drivers’ behaviors. Indeed, a TRB (Temporal Reasoning Block) has been introduced to model the causes of behaviors. The aim of this 3D-CNN is to discriminate spatio-temporal representations with attention saliency mechanisms. By assuming inputs as coarse-grained videos, the main contribution here is the proposition of a novel reasoning block composed of two layers; the first one consists of fine-grained 3D convolution and the second one allows to keep the temporal continuity.

**CNN Features Extractors.** With the availability of large-scale visual data along with optimization algorithms and powerful CPUs/GPUs, it becomes possible to train deep networks that achieve impressive performance on roughly all the challenging tasks. The obtained models are shared for the community in order to avoid training from scratch and make it easier for researchers to enhance the available models or reuse them as features extractors. For example, a CNN network that has been trained to classify road objects will output several features from the low-level to the high-level layers with increasing complexity and abstraction. Complexity, in this case, goes from pixels, blobs, circles, wheels, stuff, faces, hoods until bicycles, cars, pedestrians, and the whole scene. A number of neurons are supposed to be activated for these abstraction levels. The key feature of this is that another classification task devoted for example for the indoor or in-the-wild scenes will make use of the same low- and mid-level features that are present in all the domains.

The ImageNet [21] and the COCO [70] pre-trained models are widely considered for RBA tasks and reused as backbones for feature extraction. The (meta-)architectures of neural networks reviewed in this survey did make use of state-of-the-art backbones along with task-specific layers (for, e.g., classification or detection heads). Thus, choosing the right feature extractor is important since its properties (mainly the type of layers and number of parameters) directly affect the performance of the whole network. By barring the few examples that decouple the backbone from the meta-architecture [71], the main feature extractors used in the related works are AlexNet [21], ResNet-50 [58], VGG-16 [56], Inception-v2 [72], and InceptionResnet-v2 [73] used in the following non-exhaustive list of papers detailed above [16,17,18,32,43], respectively.

### 5.2. Deep Recurrent Neural Networks

Despite its impressive performance for tasks related to image analysis, CNNs examine only the current input and fail in handling sequential data. RNNs (Recurrent Neural Networks) however process only sequential data [74]. Composed often of a single node with internal memory, the principle of RNNs is memorizing the outputs and feeding them back as inputs and continuing to do this until predicting the output of the layer. Thus, RNNs allow saving information that has occurred in the past and looks for patterns over time and the length of the sequence. With application to ADAS and AV systems, such end-to-end machines have been recently used to automatically model non-linear discriminative representations to improve the performance of vision-based analysis tools basically related to RBA. We can categorize the related work depending on the architectures into two groups: approaches based on long short-term memory (LSTM) [75] and GRU (Gated Recurrent Units) [76].

To start, an LSTM network has been used in Ref. [17] to propose the so-called ADMD system (reviewed in Section 4) composed of four blocks to learn driving maneuvers as spatio-temporal sequences. Features that serve as an input for the predictor, i.e., LSTM network, were transferred from a CNN model (InceptionResnet-v2 [73]), as in [22], an attention map generator, and raw vehicle signals.

Similarly, a novel network architecture called TraPHic is introduced in [39] to predict road agent trajectories under complex contexts. The issue with LSTM under such circumstances is the inability to model relationships of heterogeneous road agents since the settings of an LSTM unit are independent of the others. To capture temporal dependencies of objects’ spatial coordinates, LSTMs are employed and combined with CNN to boost the learning of local objects’ relationships in space and time. In Ref. [43], a two-stream deep architecture for event proposal and prediction is proposed. The first one proposed the key-frames of the event sequences. A standard LSTM classifier is employed to predict the corresponding class among *approaching, entering, passing*. The output of this first stream, the candidate frames, is then forwarded to the second stream of prediction. The frames are here aggregated through GAP and output the event class.

Still, in the hybrid architectures, the dilated FCN detailed in the previous section of the work introduced in Ref. [50] has been combined with an LSTM network to predict driving models. The pros of this model are the ability to jointly learn segmentation and driving loss and predict the corresponding behavior. Using multi-modal cues coming from the visual and the sensor states, the LSTM network fuses all the *t* and t−1 into a single state including all the historical data from all the sensors. Typically using the same multi-modal data, the DBUS system explained earlier models temporal information through a bidirectional variant of LSTM, namely Bi-LSTM. Contrary to the usual LSTM, which is unidirectional, the Bi-LTSM runs inputs in two ways along, from t−1 to t+1 states and *vice versa*.

As for the GRU networks applied for RBA tasks, this is still a niche area. For instance, Hong et al. [51] propose a new encoder-decoder architecture where an RNN-based composed of a single GRU cell has been employed as a decoder. Unlike LSTM units, GRU has the ability to control the inputs without memorizing in-between status. This allows a less complex decoder block in this encoder-decoder architecture [51]. A more fancy unit has been proposed in Ref. [44] called GRFU (Gated Recurrent Fusion Units) aiming to learn temporal data and fusion simultaneously. Precisely, the novel gating mechanism learns a representation of every single-mode, i.e., sensor, for each instance in order to infer the best fusion strategy among Late Recurrent Summation (LRS), Early Gated Recurrent Fusion (EGRF), or Late Gated Recurrent State Fusion (LGRF).

## 6. Benchmarks: Datasets and Metrics

In this section, we are going to compare recent publicly available datasets in terms of their target tasks and diversity along with the used metrics.

### 6.1. Datasets

Unlike major computer vision tasks, ADAS and AV vision-based areas overall still lack benchmarks especially for RBA tasks. Holistic datasets refer to datasets that provide rich road and driving scene annotations. A comprehensive dataset like this makes it possible to tackle single or multiple tasks at once and urges researchers to come up with unified frameworks capable to learn high-level analysis through encoding several semantic information. The number of holistic datasets is very limited in the literature, thus we expose all the recent datasets that can be reused at least for one of the RBA components. In Table 1, we illustrate a comparison of the recent state-of-the-art datasets by detailing their scopes and cardinalities. To be aligned with the taxonomy provided earlier in the paper, the problem space corresponds to one of the RBA’s components.

**Brain4cars [48].** To the best of our knowledge, Brain4cars is the unique driving dataset that contains video sequences from inside and outside the vehicle. Speed and GPS information were acquired simultaneously with visual data coming from two views. The inside and the outside cameras capture the face motion of the driver and the view of the road ahead, respectively. To ensure the diversity of data, 10 persons were asked to drive the vehicle under fully natural settings across 2 physically different locations and different traffic conditions (for around 1899 km of sub-urban, urban, and highway environments). Better scalability should be gained if the recording has been done over more than two months to guarantee different weather conditions. A total of 700 images sequences were acquired and labeled on 4 different maneuver classes including right lane change, left lane change, right turn, and left turn and driving straight. Each sample is also annotated with the first instance the maneuver starts on. Further annotations have been provided as well regarding the lane information such as the actual lane of the vehicle and the number of lanes on the road.

**Berkeley DeepDrive Video dataset (BDDV) [50].** This dataset is one of the largest comprehensive datasets devoted to many tasks such as crowd-sourced driving behavior analysis, road objects detection, instance segmentation, and lane marking. Various driving scenarios have been acquired in different traffic settings including rural, urban, and highway areas under different weather conditions. BDDV englobes around 10k hours of video sequences, which contain in total 100k images. BDDV also contains multi-modal information acquired with another kind of sensor such as IMU, GPS, gyroscope, and magnetometer.

**Honda Research Institute Driving Dataset (HDD) [22].** This dataset is devoted to learning holistic driving scenes through the understanding of the drivers’ behaviors and the interactions with all the road agents in relation to the context of the scene. The scale of the dataset is large enough to allow training and validation for deep neural networks. Precisely, HDD has been collected in the San Francisco Bay Area of over 137 driving sessions and involves 104 h of videos acquired with a road-ahead camera along with a multi-sensor composed of LiDAR, IMU, GPS, and CAN bus. Following a novel annotation protocol, the authors have succeeded in proposing a frame-level description of four layers for each sample, i.e., Goal-oriented action, Stimulus-driven action, Cause, and Attention. The number of classes of each level is different. For the Goal-oriented action, 11 classes have been considered including intersection passing, left turn, right turn, crosswalk passing, left lane change, right lane change, merge, right lane branch, railroad passing, and u-turn. For the second level, annotation of Stimulus-driven includes just two classes: stop and derivate. As for the following Cause layer, five annotations have been presented: sign, congestion, traffic light, parked car, and pedestrian. The fourth Attention layer includes 12 categories: crossing vehicle, red light, crossing pedestrian, cut-in, merging vehicle, sign, on-road bicyclist, yellow light, parked vehicle, road work, on-road motorcyclist, pedestrian near ego lane.

**Driving Behavior Net (DBNet) [47].** Similarly to HDD, DBNet deals with learning driving circumstances and policies. The major advantage of DBNet is the consideration of 3D point clouds and depth information with a range of 70 m and a resolution up to 2 cm. For this purpose, two different kinds of LiDAR sensors have been exploited to record 3D point clouds. The coverage of DBNet goes for 100 km, which allows the collection of seven multi-modal sets of different driving scenes. Three kinds of data have been recorded: (i) point clouds, (ii) color videos with the corresponding (iii) vehicle’ speed and steering angle information. The data have been recorded under challenging contexts with traffic conditions (normal and crowd flows). Overall, the dataset contains labels for around 32 footbridges, 160 traffic lights, 500 road signs, and 1500 cars.

**Argoverse [42].** The authors proposed to support mid-level and high-level AV vision-based tasks such as trajectories prediction, 3D tracking, and map-based perception. By means of a platform equipped with synchronized sensors composed of LiDAR, 360° and stereo cameras, the Argoverse data is acquired in the coverage area of 300 km in Pittsburgh and Miami cities, which have distinct building architectures and weather conditions. Besides standard 2D and 3D information, three different kinds of maps have been generated (cf. [42] for further details). The proposed annotations contain 3D tracks of 10,572 objects dispersed over 100 sequences for a duration up to 60 s.

**TRAF [39].** Dense and heterogeneous road traffics have been considered by the TRAF dataset. The scale of the dataset is not very large since only 50 sequences enclosing around 12,400 frames have been collected under urban conditions. The advantage of the samples of this dataset is that they cover eight road agents against three for the NGSIM dataset [40], mainly *‘rickshaw’, ‘truck’, ‘pedestrian’, ‘motorcycle’, ‘scooter’, ‘car’, ‘truck’, and ‘bus’*. Thus, the density of the TRAF is quite high and on average we can find 15 motorized and non-motorized vehicles and 5 pedestrians per frame. Annotations include trajectories organized as 2D coordinates of each frame, the category of the agent, and its corresponding ID. Visual scenes have been classified using a two-viewpoint and other cues like motion, daytime, and density level.

**Intersection Dataset [38].** To evaluate the proposed DROGON framework, introduced in Section 4, the Intersection dataset has been presented to deal with the issue of trajectories prediction in high dynamic scenarios, basically, at four-way road intersection with cluttered traffic in San Francisco Bay Area at four different regions. A total of 213 intersection scenes have been acquired using multi-sensor, which have in total $59.4\times 10^{3}$ frames. Annotations made for the intersection dataset are the most extensive compared to other datasets and consist of the category of road agents (8 classes available) and their corresponding trajectories ID, 3D bounding boxes, intentional zone, odometry of the AV, heading angles, point clouds of 360° coverage, and driveable road area.

**Joint Attention in Autonomous Driving (JAAD) [18].** This is considered as the first dataset devoted to the situational awareness understanding of the AV with regards to pedestrians at the crossing points. Specifically, JAAD includes behavioral and contextual information along with tracking information of 337,000 detected pedestrians from 346 video sequences collected in Europe and North America. By means of the BORIS tool [77], behavioral annotations have been provided to capture the action of both driver and the facing pedestrians. The driver’s action label includes Moving fast/slow as the first state and *Slow down/Speed up* as a response to the surroundings. The pedestrians’ actions are classified into three sets: (i) Precondition: activities before crossing such as standing, or moving fast (ii) Attention: gestural and temporal cues which serve to understand whether the person is aware of the approaching (ego-)vehicle and (iii) Response: to check whether the crossing pedestrian is reacting to the behavior of the approaching vehicle through identifying one of the following elementary action/gesture: Clear path, Speed up, Slow down, Hand Gesture, Nod, and Stop. Moreover, demographic annotations are provided for each detected person such as age, class, and gender. Along with behavioral labels, scene contextual information is provided. Four kinds of information are proposed: (i) Configurations of the road: parking lot or the number of lanes. (ii) Traffic signals: such as traffic lights, stop signs, or pedestrian signs. (iii) Weather condition, and (iv) Time of day: to describe the lighting conditions.

**Honda Research Institute-Advice Dataset (HAD) [78]**. Taking profit of their HDD dataset, Honda research institute proposed HAD as a sub-set of 5600 videos selected from HDD with a completely new configuration. The object of HAD is to allow better advisable frameworks for AVs with human-to-vehicle advice annotations. Each sample of the dataset has 4 to 5 action tags and 3 to 4 attention tags. For example, *‘The driver change the lane from left to right’* in an action label and its corresponding annotation label is *‘Work on progress on the road’*. We refer the reader to the HDD description detailed earlier in this section for further information regarding the modality and diversity of its re-annotated subset, i.e., HAD.

**Occluded Vehicle Dataset (OVD) [16].** In an original way, OVD combines real and synthetic images for segmentation with vehicle appearance recovery. Synthetic data have been leveraged from the Cars dataset [79] and real data have been extracted from the COCO [70] and Cityscapes [80] datasets. Particularly, images of cars included in the Cars dataset are unoccluded in most of the cases, so to make them usable for this task, cropped objects (pedestrians, vehicles, etc.) from COCO and Cityscapes are placed on the Cars instances, which have clear ground truth. Overall, the dataset contains 34,100 images. Besides, 4 video sequences and 100 images have been collected and manually labeled to enrich the diversity of the dataset.

**nuTonomy scenes (nuScenes) [81].** Recently published in late 2019, nuScenes is the largest dataset devoted for mid-level scene understanding, mainly focusing on 3D detection and tracking, which surpasses 7 times the benchmark KITTI [36] in terms of diversity and 100 times in terms of scalability. The platform used to collect this dataset is fully equipped with multi-modal sensors (RGB, LiDAR, and RaDAR) with full 360° point-of-view. Regarding its scalability, it comprises 1000 image sequences labeled with 3D bounding boxes. For each image, annotations of the detected objects classes are provided, which enclose the attributes of the road objects in addition to their semantic categories. As an example for the values of the attributes, we can find the exact pedestrian pose (such as ‘walking’, ‘approaching’) or the vehicle state (such as ‘parked’, ‘standing’). Numerically, 23 objects categories have been considered with 8 different attributes. What makes nuScenes very unique is its huge diversification coming from the different acquisition sites ranging from nature to industrial under different time and weather conditions collected over a whole year. Table 1 summarize the recent RBA datasets.

### 6.2. Metrics

To objectively assess the effectiveness of RBA components, many metrics have been imported from other computer vision tasks, and others have been introduced to evaluate a specific model’s performance. We start with the presentation of traditional criteria that have been re-used for most of the RBA scenarios followed by the novel metrics introduced to tackle the newly introduced tasks.

Traditionally, accuracy, F1-score, recall, and precision have been commonly employed to assess the performance of the proposed approaches. Aside from a few number of works that did reuse these metrics [16,48], new criteria have been proposed by the RBA community to discern the performance of a given model designed for a new tasks and/or dataset configurations.

During the last decade, Average Precision (AP) was presented initially to the computer vision community during the PASCAL Visual Object Classes (VOC) Challenge 2007 [82] and since then it has been widely employed to assess the performance of RBA approaches. For example, in Refs. [18,50] to evaluate the driving model, a per-frame AP has been computed for each behavior categories through ranking frames with respect to a confidence score. The mean AP (mAP) is then computed over all the available categories.

For mid-level RBA understanding tasks, such as detection, tracking, segmentation, and appearance recovery, common measures such as IoU (Intersection over Union), MOTP (Multiple Object Tracking Precision), and MOTA (Multiple Object Tracking Accuracy) have been extensively employed. IoU quantifies the localization of objects, however, MOTP and MOTA consider intuitively the strength of a given tracker, in terms of precision and accuracy, respectively. For the tracking metrics, an extension of the two latter metrics has been introduced in Ref. [83], i.e., AMOTA and AMOTP, which correspond to an average of MOTA and MOTP, respectively, over the recall thresholds. According to the baselines drawn over challenging benchmarks, the above-listed metrics were not consistent for domains with frequent temporal object occlusions, like roads, where the tracker might miss information on tracked objects for a significant time slot. Thus, in [81], Caesar et al. bring out **TID (Track Initialization Duration)** and **LGD (Longest Gap Duration)**, which are consistent for AV. Expressly, TID helps in providing better initialization through quantifying the time slot between the tracker’s start time and the first detection time. LGD however measures the longest time slot of a detection gap/occlusion. For a given sequence, the final TID and LGD are then averaged over all the available tracks. As for the detection metrics, traditional metrics like mAP and IoU are not always able to apprehend errors related to velocity and attribute estimation like in the case of nuScenes. To deal with this, **NDS (NuScenes Detection Score)** is proposed to solve this issue and by encapsulating mAP and TP (True Positive) for five different cues with different weights. So, NDS is able to quantify not only the detection’s performance but also its quality with regards to velocity, orientation, location, attributes, and size.

For segmentation and appearance recovery tasks, aside from traditional metrics, i.e., precision, recall, F1-score, IoU, per-pixel L1−error, and per-pixel L2−error, we witnessed the introduction of new sophisticated criteria. **ICP (Inception Conditional Probability)** and **SS (Segmentation Score)** originally introduced and used in [84] and [16], respectively, both aim at assessing the generation/recovery quality rather than the diversity. The intuition is to classify the generated image region of the occluded-segmented road object correctly using a model built and trained using real data.

As for tasks involving trajectories forecasting [38,39], in [85] Alahi et al. propose to measure **Average Distance Error (ADE)** and **Final Distance Error (FDE)** at a given time slot in meters to estimate the performance of the predict trajectories of road agents. ADE is computed as the RMSE (Root Mean Square Error) between all the real and the predicted positions and FDE corresponds to the RMSE of the final predicted location and its corresponding real location at the end of the trajectories. Qualitative evaluation has been also used to compare the obtained trajectories of various approaches plotted on derivable regions.

Based on language modeling, a new criterion has been introduced in Ref. [50] called **Driving Perplexity (DP)** to evaluate the driving behavior. It corresponds to the exponent of the entropy of all predicted events in a given sequence. Thus, the DP value is between zero and one, and the smaller the value is, the more accurate the prediction is [28].

## 7. Discussion and Future Directions

In this review, we focused on holistic on-road scene behaviors analysis through identifying all related tasks and their corresponding challenges. To conclude this paper, we will discuss in this section the open challenges and general aspects that have an impact on the key steps of the AV and ADAS vision-based standards.

**Deep learning for perception.** To allow safe on-road navigation, we need to provide a broad description of the AV’ surroundings. Traditional machine learning-based approaches did suffer from many issues in the traffic environment-related mainly to (i) the uncontrolled conditions of the visual domain (such as gradual and sudden illumination changes, noises due to extreme weathers) and (ii) the large diversity of non-static road agents (such as the huge variation of motions, poses, and textures). We believe that deep learning-based approaches have succeed to overcome most of these issues. Details regarding the progress of deep architectures exposed in this review prove that networks like CNN, GCN, FCN, and LSTM are capable to build and training models that are more robust and especially able to better generalize the circumstances, i.e., models generated are more able to deal with new/unseen data issues from the same modality space as the one used to build the model. For e.g., many of the reviewed meta-architectures evaluated over AV datasets did make use of backbones pre-trained from wild and indoor datasets. However, one of the biggest barriers of deep neural networks is the inability to adapt models for different “domains”, i.e., modalities. In fact, most of the exposed RBA datasets are multi-modal (include various kinds of data such as RGB, Depth, LiDAR, RaDAR, GPS, etc.), but most of the endeavors did propose a single-mode or multi-mode framework where their networks settings are inherited from/to models belonging to the same domain. To better use the available multi-modal datasets, the ITS community needs to go for domain adaptation techniques [86] in order to propose new algorithms aiming to adapt the parameters of a given network developed for a specific dataset to a different one. Another issue related to the employment of deep learning is the computational cost. In fact, even though such solutions are now available on the market [87], research is still lacking proposals to enable the deployment of resource-constrained vehicles.

**Spatio-temporal modeling.** The reported state-of-the-art shows that the best spatio-temporal fit to the RBA data is the hybrid structures of CNNs and LSTMs. The principle is to extract spatial features for each instance and feed them into the LSTM layer, which is able to capture the temporal representation. Through the literature evaluation, we can note that the major issues are related to computational time. Indeed, common Nvidia graphic cards, which are the ultimate deep learning hardware, perform better in the context of spatial/2D data owing to its powerful paradigm of parallelism and speed. Thus, CNNs are the best fit to the available computational resources and, contrary to LSTMs, which are not hardware friendly, they handle sequential series, which will decrease the speed of training and inference. In addition, due to the large number of parameters a single node of LSTM has, the overfitting problem and sensitivity to random initialization of weight is an impediment for such hybrid architectures. Despite the success of 3D-CNN and attention mechanisms that prevail LSTMs in many vision-based tasks [88,89,90], there are few proposals introduced to exploit them with regards to RBA tasks, which will enable them to get the end-to-end framework, transferable model, and better spatial correlation.

**“Blessing” and “Curse” of data.** The introduction of new datasets each time opens new perspectives and challenges, which exhibits the advances of building robust RBA components. Recent datasets, raised up in the benchmarks section of this review, cover all the best properties of the vision dataset, i.e., scale, modalities, and diversities. This choice is driven by the increasing need for machine/deep learning algorithms where training a network for example is tied to a large scale of training data. It is obvious that the quality of data will impact directly the learning process, however, it is not that easy to get reliable data settings with high dimensions. In fact, datasets are annotated manually or using labeling tools like LabelMe [91] and ELAN [92]. Large-scale datasets are indeed sparsely annotated and further processing should be done to discern unlabeled data. This issue has not been taken into account with RBA benchmarks, despite its importance in tackling their incompleteness. One possible direction might be the use of pre-trained models to check the reliability of annotation or use human-level intuition like the part-aware approach proposed in Ref. [93].

**RBA corner cases.** Towards holistic understanding, approaches need to cover all the usual and critical situations under the same framework. As reviewed earlier, the recent state-of-the-art succeeded to introduce tasks including various on-road situations, however, there is still a lot of work to be done. To do so, a new task called corner case detection has been recently introduced, which aims at detecting unusual circumstances [94]. However, until now, the corner cases task is still a niche area. Future directions may include the generalization of deep neural networks in order to learn hazardous situations that might lead to crashes, for example.

**Explainable and interpretable models.** With the increasing use of revolutionary deep neural networks, RBA solutions have shown an impressive performance in short term, owing to the ease of design and development of such models. Despite their apparent implementation simplicity, these algorithms are composed of black boxes that disable valid explanations for the proposed solutions and hinder end-user confidence for road safety matters. In fact, within the proposed RBA models, it is difficult to draw a prediction as to which representations are crucial. Despite their straightforward mathematical formalization, the comprehension of how the output was reached is enigmatic since many heterogeneous nodes are *interacting* and let loose on the feed data in order to build the final result. Ref. [10] tried to put forward the use of secure of deep learning architectures, but this topic is still lacking unified formalization for understandable RBA solutions to end users [95].

## 8. Conclusions

Remarkable achievements have been made in the RBA area over the last decade [1,2,3,4,5,6,7,8,9,10,11,12,13,14,15,16,17,18,19,20,21,22,23,24,25,26,27,28,29,30,31,32,33,34,35,36,37,38,39,40,41,42,43,44,45,46,47,48,49,50,51,52,53,54,55,56,57,58,59,60,61,62,63,64,65,66,67,68,69,70,71,72,73,74,75,76,77,78,79,80,81,82,83,84,85,86,87,88,89,90,91,92,93,94,95,96,97,98,99,100,101,102,103,104,105,106,107,108,109,110,111,112,113,114,115,116,117,118,119,120,121,122,123,124,125,126,127,128,129,130,131,132,133,134]. This review paper provides the first attempt to draw ITS researchers’ attention to this field and its potential impact on road safety to all the agents (drivers, pedestrians, stuffs, etc.). Even though many related tasks have been introduced since the early time, the RBA area has not been defined appropriately before. To do so, we first investigated the complementary relationships between many elementary tasks that we define as components of the RBA paradigm, in order to achieve a comprehensive understanding of the approaches and techniques. The tasks identified (situational awareness, driver-road interaction, road scene understanding, trajectories forecast, driving activities, and status analysis) were discussed separately and then common concepts and links were stressed through reviewing milestones and recent state-of-the-art. Holistic approaches that were ntroduced recently have been well assessed. Next, deep-leaning based solutions have been presented with a specific taxonomy, which can help practitioners in selecting their best-fit architecture. Finally, a discussion of the overall approaches was elaborated, which allows us to find future directions. In summary, this paper presents the first survey of RBA-related work without overlap with existing reviews elaborated on elementary topics and reports the recent advances in the field.

## Figures and Tables

**Table 1:** Summary of recent RBA datasets. From 1 to 6, the PS (Problem Space) ID refers to situational awareness (ID = 1), driver-road interaction (ID = 2), road scene understanding (ID = 3), trajectories forecast (ID = 4), driving activities and status analysis (ID = 5), and holistic understanding (ID = 6).

Dataset	Year	PS #ID	Sensor Setup	Location	Traffic Condition	Hours and/or Distance	#Sequences	#Frames
**nuScenes ** **[81]**	2019	3	Camera RADAR LiDAR GPS IMU	Boston and Singapore	Urban	55 h	1 k	1.4 M
**Argoverse ** **[42]**	2019	3 4	Camera Stereo LiDAR IMU	Pittsburgh and Miami	Urban	300 km	100	-
**TRAF** **[39]**	2019	4 5	Camera 3D sensor Motion- sensor	Asian cities	Urban	-	50	12.4 k
**Inter-** **section** **[38]**	2019	2 3 4 5 6	Camera LiDAR GPS	San Francisco Bay Area	Urban	-	213	59.4 k
**OVD [16]**	2019	3	Camera	-	-	-	4+	34.1 k
**HAD [78]**	2019	1	Camera LiDAR GPS IMU CAN	San Francisco Bay Area	Suburban Urban High way	32 h	5.6 k	-
**HDD [22]**	2018	6	Camera LiDAR GPS IMU CAN	San Francisco Bay Area	Suburban, urban and highway	104 h	-	-
**DBNet** **[47]**	2018	2 5	Camera LiDAR	-	Local route Boulevard Primary rd. Mountain roads School areas	20 h 100 km	-	-
**BDDV** **[50]**	2017	3	Camera GPS IMU Gyroscope Magne- tometer	US cities	Cities Highways Rural areas	10 k hours	-	100 k
**JAAD** **[18]**	2017	1 6	Camera	North America and Europe	-	-	346	82 k
**Brain4-** **Cars** **[48]**	2015	5 6	Camera GPS Speed- logger	Two US states	Suburban Urban Highway	1899 km	700	2 M

## Data Availability

Not applicable.

## Abbreviations

The following abbreviations are used in this manuscript:

ADAS	Advanced Driver Assistance System
AV	Autonomous Vehicles
RBA	Road Behavior Understanding
ASIRT	Association for Safe International Road Travel
ITS	Intelligent Transportation System
CNN	Convolutional Neural Network
JAAD	Joint Attention in Autonomous Driving
HDD	Honda Research Institute Driving Dataset
GCN	Graph Convolutional Networks
mAP	mean Average Precision
RNN	Recurrent Neural Network
HMM	Hidden Markov Model
CA	Class Accuracy
MIoU	Mean Intersection over Union
OVD	Occluded Vehicle Dataset
ICP	Inception Conditional Probability
SS	Segmentation Score
ADE	Average Distance Error
FDE	Final Distance Error
LSTM	Long Short-Term Memory
DBUS	Driving Behavior Understanding System
ADMD	Attention-driven Driving Maneuver Detection
ILSVRC	ImageNet Large Scale Visual Recognition Challenge
GAP	Global Average Pooling
GCN	Graph Convolutional Networks
FCN	Fully Convolutional Networks
FC	Fully Connected
MLP	Multi Layer Perceptron
RNN	Recurrent Neural Networks
GRU	Gated Recurrent Units
GRFU	Gated Recurrent Fusion Units
LRS	Late Recurrent Summation
EGRF	Early Gated Recurrent Fusion
LGRG	Late Gated Recurrent State Fusion
BDDV	Berkeley DeepDrive Video dataset
DBNet	Driving Behavior Net
HAD	Honda Research Institute-Advice Dataset
nuScenes	nuTonomy scenes
AP	Average Precision
VOC	Visual Object Classes
IoU	Intersection over Union
MOTP	Multiple Object Tracking Precision
MOTA	Multiple Object Tracking Accuracy
TID	Track Initialization Duration
LGD	Longest Gap Duration
NDS	NuScenes Detection Score
TP	True Positive
SS	Segmentation Score
FDE	Final Distance Error
RMSE	Root Mean Square Error
DP	Driving Perplexity

## References

[1] Xique I.J., Buller W., Fard Z.B., Dennis E., Hart B. Evaluating Complementary Strengths and Weaknesses of ADAS Sensors. Proceedings of the 2018 IEEE 88th Vehicular Technology Conference (VTC-Fall).

[2] Hu H.N., Cai Q.Z., Wang D., Lin J., Sun M., Krahenbuhl P., Darrell T., Yu F. Joint Monocular 3D Vehicle Detection and Tracking. Proceedings of the IEEE International Conference on Computer Vision.

[3] Bresson G., Alsayed Z., Yu L., Glaser S. (2017). Simultaneous localization and mapping: A survey of current trends in autonomous driving. IEEE Trans. Intell. Veh..

[4] Santhosh K.K., Dogra D.P., Roy P.P. (2018). Temporal unknown incremental clustering model for analysis of traffic surveillance videos. IEEE Trans. Intell. Transp. Syst..

[5] Luckow A., Cook M., Ashcraft N., Weill E., Djerekarov E., Vorster B. Deep learning in the automotive industry: Applications and tools. Proceedings of the 2016 IEEE International Conference on Big Data (Big Data).

[6] Pouyanfar S., Sadiq S., Yan Y., Tian H., Tao Y., Reyes M.P., Shyu M.L., Chen S.C., Iyengar S. (2019). A survey on deep learning: Algorithms, techniques, and applications. ACM Comput. Surv. (CSUR).

[7] Talpaert V., Sobh I., Kiran B.R., Mannion P., Yogamani S., El-Sallab A., Perez P. (2019). Exploring applications of deep reinforcement learning for real-world autonomous driving systems. arXiv.

[8] Chao Q., Bi H., Li W., Mao T., Wang Z., Lin M.C., Deng Z. (2019). A survey on visual traffic simulation: Models, evaluations, and applications in autonomous driving. Comput. Graph. Forum.

[9] Sivaraman S., Trivedi M.M. (2013). Looking at vehicles on the road: A survey of vision-based vehicle detection, tracking, and behavior analysis. IEEE Trans. Intell. Transp. Syst..

[10] Liu Y., Ma L., Zhao J. (2019). Secure Deep Learning Engineering: A Road Towards Quality Assurance of Intelligent Systems. Proceedings of the International Conference on Formal Engineering Methods.

[11] Kendall A., Hawke J., Janz D., Mazur P., Reda D., Allen J.M., Lam V.D., Bewley A., Shah A. Learning to drive in a day. Proceedings of the 2019 International Conference on Robotics and Automation (ICRA).

[12] Ngai D.C.K., Yung N.H.C. (2011). A multiple-goal reinforcement learning method for complex vehicle overtaking maneuvers. IEEE Trans. Intell. Transp. Syst..

[13] Wang P., Chan C.Y. Formulation of deep reinforcement learning architecture toward autonomous driving for on-ramp merge. Proceedings of the 2017 IEEE 20th International Conference on Intelligent Transportation Systems (ITSC).

[14] Adadi A., Berrada M. (2018). Peeking inside the black-box: A survey on Explainable Artificial Intelligence (XAI). IEEE Access.

[15] Samek W., Wiegand T., Müller K.R. (2017). Explainable artificial intelligence: Understanding, visualizing and interpreting deep learning models. arXiv.

[16] Yan X., Wang F., Liu W., Yu Y., He S., Pan J. Visualizing the Invisible: Occluded Vehicle Segmentation and Recovery. Proceedings of the IEEE International Conference on Computer Vision.

[17] Peng X., Zhao A., Wang S., Murphey Y.L., Li Y. Attention-Driven Driving Maneuver Detection System. Proceedings of the 2019 International Joint Conference on Neural Networks (IJCNN).

[18] Rasouli A., Kotseruba I., Tsotsos J.K. Are they going to cross? A benchmark dataset and baseline for pedestrian crosswalk behavior. Proceedings of the IEEE International Conference on Computer Vision.

[19] Kooij J.F.P., Schneider N., Flohr F., Gavrila D.M. (2014). Context-based pedestrian path prediction. Proceedings of the European Conference on Computer Vision.

[20] Meinecke M.M., Obojski M., Gavrila D., Marc E., Morris R., Tons M., Letellier L. (2003). Strategies in terms of vulnerable road user protection. EU Proj. SAVE-U Deliv. D.

[21] Krizhevsky A., Sutskever I., Hinton G.E. Imagenet classification with deep convolutional neural networks. Proceedings of the Advances in Neural Information Processing Systems.

[22] Ramanishka V., Chen Y.T., Misu T., Saenko K. Toward driving scene understanding: A dataset for learning driver behavior and causal reasoning. Proceedings of the IEEE Conference on Computer Vision and Pattern Recognition.

[23] Li C., Meng Y., Chan S.H., Chen Y.T. (2019). Learning 3D-aware Egocentric Spatial-Temporal Interaction via Graph Convolutional Networks. arXiv.

[24] Wu J., Wang L., Wang L., Guo J., Wu G. Learning Actor Relation Graphs for Group Activity Recognition. Proceedings of the IEEE Conference on Computer Vision and Pattern Recognition.

[25] Oliver N., Pentland A.P. Graphical models for driver behavior recognition in a smartcar. Proceedings of the IEEE Intelligent Vehicles Symposium 2000 (Cat. No. 00TH8511).

[26] Singh D., Mohan C.K. (2018). Deep spatio-temporal representation for detection of road accidents using stacked autoencoder. IEEE Trans. Intell. Transp. Syst..

[27] Hu X., Xu X., Xiao Y., Chen H., He S., Qin J., Heng P.A. (2018). SINet: A scale-insensitive convolutional neural network for fast vehicle detection. IEEE Trans. Intell. Transp. Syst..

[28] Chen B., Gong C., Yang J. (2018). Importance-aware semantic segmentation for autonomous vehicles. IEEE Trans. Intell. Transp. Syst..

[29] Aly M. Real time detection of lane markers in urban streets. Proceedings of the 2008 IEEE Intelligent Vehicles Symposium.

[30] Gong J., Jiang Y., Xiong G., Guan C., Tao G., Chen H. The recognition and tracking of traffic lights based on color segmentation and camshift for intelligent vehicles. Proceedings of the 2010 IEEE Intelligent Vehicles Symposium.

[31] Gavrila D.M., Giebel J., Munder S. Vision-based pedestrian detection: The PROTECTOR system. Proceedings of the IEEE Intelligent Vehicles Symposium.

[32] Zhou W., Lv S., Jiang Q., Yu L. (2019). Deep Road Scene Understanding. IEEE Signal Process. Lett..

[33] Fauqueur J., Brostow G., Cipolla R. Assisted video object labeling by joint tracking of regions and keypoints. Proceedings of the 2007 IEEE 11th International Conference on Computer Vision, Rio de Janeiro.

[34] Chen L.C., Papandreou G., Kokkinos I., Murphy K., Yuille A.L. (2017). Deeplab: Semantic image segmentation with deep convolutional nets, atrous convolution, and fully connected crfs. IEEE Trans. Pattern Anal. Mach. Intell..

[35] Brust C.A., Sickert S., Simon M., Rodner E., Denzler J. (2015). Convolutional Patch Networks with Spatial Prior for Road Detection and Urban Scene Understanding. arXiv.

[36] Geiger A., Lenz P., Urtasun R. Are we ready for autonomous driving? The kitti vision benchmark suite. Proceedings of the 2012 IEEE Conference on Computer Vision and Pattern Recognition.

[37] Frohlich B., Rodner E., Denzler J. A fast approach for pixelwise labeling of facade images. Proceedings of the 2010 20th International Conference on Pattern Recognition.

[38] Choi C., Patil A., Malla S. (2019). Drogon: A causal reasoning framework for future trajectory forecast. arXiv.

[39] Chandra R., Bhattacharya U., Bera A., Manocha D. Traphic: Trajectory prediction in dense and heterogeneous traffic using weighted interactions. Proceedings of the IEEE Conference on Computer Vision and Pattern Recognition.

[40] US Department of Transportation (2007). NGSIM—Next Generation Simulation. https://ops.fhwa.dot.gov/trafficanalysistools/ngsim.htm.

[41] Chandra R., Bhattacharya U., Mittal T., Li X., Bera A., Manocha D. (2019). GraphRQI: Classifying Driver Behaviors Using Graph Spectrums. arXiv.

[42] Chang M.F., Lambert J., Sangkloy P., Singh J., Bak S., Hartnett A., Wang D., Carr P., Lucey S., Ramanan D. Argoverse: 3D Tracking and Forecasting with Rich Maps. Proceedings of the IEEE Conference on Computer Vision and Pattern Recognition.

[43] Narayanan A., Dwivedi I., Dariush B. (2019). Dynamic Traffic Scene Classification with Space-Time Coherence. arXiv.

[44] Narayanan A., Siravuru A., Dariush B. (2019). Temporal Multimodal Fusion for Driver Behavior Prediction Tasks using Gated Recurrent Fusion Units. arXiv.

[45] Taha A., Chen Y.T., Misu T., Davis L. (2019). In Defense of the Triplet Loss for Visual Recognition. arXiv.

[46] Guangyu Li M., Jiang B., Che Z., Shi X., Liu M., Meng Y., Ye J., Liu Y. DBUS: Human Driving Behavior Understanding System. Proceedings of the IEEE International Conference on Computer Vision Workshops.

[47] Chen Y., Wang J., Li J., Lu C., Luo Z., Han X., Wang C. LiDAR-Video Driving Dataset: Learning Driving Policies Effectively. Proceedings of the IEEE Conference on Computer Vision and Pattern Recognition (CVPR).

[48] Jain A., Koppula H.S., Raghavan B., Soh S., Saxena A. Car that knows before you do: Anticipating maneuvers via learning temporal driving models. Proceedings of the IEEE International Conference on Computer Vision.

[49] Bengio Y., Frasconi P. An input output HMM architecture. Proceedings of the Advances in Neural Information Processing Systems.

[50] Xu H., Gao Y., Yu F., Darrell T. End-to-end learning of driving models from large-scale video datasets. Proceedings of the IEEE Conference on Computer Vision and Pattern Recognition.

[51] Hong J., Sapp B., Philbin J. Rules of the Road: Predicting Driving Behavior with a Convolutional Model of Semantic Interactions. Proceedings of the IEEE Conference on Computer Vision and Pattern Recognition.

[52] LeCun Y., Bottou L., Bengio Y., Haffner P. (1998). Gradient-based learning applied to document recognition. Proc. IEEE.

[53] Mauri A., Khemmar R., Decoux B., Ragot N., Rossi R., Trabelsi R., Boutteau R., Ertaud J.Y., Savatier X. (2020). Deep learning for real-time 3D multi-object detection, localisation, and tracking: Application to smart mobility. Sensors.

[54] Liu W., Anguelov D., Erhan D., Szegedy C., Reed S., Fu C.Y., Berg A.C. (2016). Ssd: Single shot multibox detector. Proceedings of the European Conference on Computer Vision.

[55] Redmon J., Divvala S., Girshick R., Farhadi A. You only look once: Unified, real-time object detection. Proceedings of the IEEE Conference on Computer Vision and Pattern Recognition.

[56] Simonyan K., Zisserman A. (2014). Very deep convolutional networks for large-scale image recognition. arXiv.

[57] Szegedy C., Liu W., Jia Y., Sermanet P., Reed S., Anguelov D., Erhan D., Vanhoucke V., Rabinovich A. Going deeper with convolutions. Proceedings of the IEEE Conference on Computer Vision and Pattern Recognition.

[58] He K., Zhang X., Ren S., Sun J. Deep residual learning for image recognition. Proceedings of the IEEE Conference on Computer Vision and Pattern Recognition.

[59] Chollet F. Xception: Deep learning with depthwise separable convolutions. Proceedings of the IEEE Conference on Computer Vision and Pattern Recognition.

[60] Kipf T.N., Welling M. Semi-Supervised Classification with Graph Convolutional Networks. Proceedings of the International Conference on Learning Representations (ICLR).

[61] He K., Gkioxari G., Dollár P., Girshick R. Mask r-cnn. Proceedings of the IEEE International Conference on Computer Vision.

[62] Dai J., Li Y., He K., Sun J. R-fcn: Object detection via region-based fully convolutional networks. Proceedings of the Advances in Neural Information Processing Systems.

[63] Maggiori E., Tarabalka Y., Charpiat G., Alliez P. Can semantic labeling methods generalize to any city? The inria aerial image labeling benchmark. Proceedings of the 2017 IEEE International Geoscience and Remote Sensing Symposium (IGARSS).

[64] Long J., Shelhamer E., Darrell T. Fully convolutional networks for semantic segmentation. Proceedings of the IEEE Conference on Computer Vision and Pattern Recognition.

[65] Zhang X., Zhou X., Lin M., Sun J. Shufflenet: An extremely efficient convolutional neural network for mobile devices. Proceedings of the IEEE Conference on Computer Vision and Pattern Recognition.

[66] Siam M., Gamal M., Abdel-Razek M., Yogamani S., Jagersand M., Zhang H. A comparative study of real-time semantic segmentation for autonomous driving. Proceedings of the IEEE Conference on Computer Vision and Pattern Recognition Workshops.

[67] Yi L., Su H., Guo X., Guibas L.J. Syncspeccnn: Synchronized spectral cnn for 3d shape segmentation. Proceedings of the IEEE Conference on Computer Vision and Pattern Recognition.

[68] Xu H., Das A., Saenko K. R-c3d: Region convolutional 3d network for temporal activity detection. Proceedings of the IEEE International Conference on Computer Vision.

[69] Liu Y.C., Hsieh Y.A., Chen M.H., Yang C.H.H., Tegner J., Tsai Y.C.J. (2019). Interpretable Self-Attention Temporal Reasoning for Driving Behavior Understanding. arXiv.

[70] Lin T.Y., Maire M., Belongie S., Hays J., Perona P., Ramanan D., Dollár P., Zitnick C.L. (2014). Microsoft coco: Common objects in context. Proceedings of the European Conference on Computer Vision.

[71] Lee S., Kim J., Oh T.H., Jeong Y., Yoo D., Lin S., Kweon I.S. (2019). Visuomotor Understanding for Representation Learning of Driving Scenes. arXiv.

[72] Szegedy C., Vanhoucke V., Ioffe S., Shlens J., Wojna Z. Rethinking the inception architecture for computer vision. Proceedings of the IEEE Conference on Computer Vision and Pattern Recognition.

[73] Szegedy C., Ioffe S., Vanhoucke V., Alemi A.A. Inception-v4, inception-resnet and the impact of residual connections on learning. Proceedings of the Thirty-First AAAI Conference on Artificial Intelligence.

[74] Lipton Z.C., Berkowitz J., Elkan C. (2015). A critical review of recurrent neural networks for sequence learning. arXiv.

[75] Zhu X., Sobihani P., Guo H. Long short-term memory over recursive structures. Proceedings of the International Conference on Machine Learning.

[76] Cho K., Van Merriënboer B., Gulcehre C., Bahdanau D., Bougares F., Schwenk H., Bengio Y. (2014). Learning phrase representations using RNN encoder-decoder for statistical machine translation. arXiv.

[77] Friard O., Gamba M. (2016). BORIS: A free, versatile open-source event-logging software for video/audio coding and live observations. Methods Ecol. Evol..

[78] Kim J., Misu T., Chen Y.T., Tawari A., Canny J. Grounding Human-To-Vehicle Advice for Self-Driving Vehicles. Proceedings of the IEEE Conference on Computer Vision and Pattern Recognition (CVPR).

[79] Krause J., Stark M., Deng J., Fei-Fei L. 3d object representations for fine-grained categorization. Proceedings of the IEEE International Conference on Computer Vision Workshops.

[80] Cordts M., Omran M., Ramos S., Rehfeld T., Enzweiler M., Benenson R., Franke U., Roth S., Schiele B. The cityscapes dataset for semantic urban scene understanding. Proceedings of the IEEE Conference on Computer Vision and Pattern Recognition.

[81] Caesar H., Bankiti V., Lang A.H., Vora S., Liong V.E., Xu Q., Krishnan A., Pan Y., Baldan G., Beijbom O. (2019). nuscenes: A multimodal dataset for autonomous driving. arXiv.

[82] Everingham M., Van Gool L., Williams C.K.I., Winn J., Zisserman A. The PASCAL Visual Object Classes Challenge 2007 (VOC2007) Results. http://www.pascal-network.org/challenges/VOC/voc2007/workshop/index.html.

[83] Weng X., Kitani K. (2019). A baseline for 3d multi-object tracking. arXiv.

[84] Salimans T., Goodfellow I., Zaremba W., Cheung V., Radford A., Chen X. Improved techniques for training gans. Proceedings of the Advances in Neural Information Processing Systems.

[85] Alahi A., Goel K., Ramanathan V., Robicquet A., Fei-Fei L., Savarese S. Social lstm: Human trajectory prediction in crowded spaces. Proceedings of the IEEE Conference on Computer Vision and Pattern Recognition.

[86] Ma X., Zhang T., Xu C. (2019). Deep Multi-Modality Adversarial Networks for Unsupervised Domain Adaptation. IEEE Trans. Multimed..

[87] (2020). PRESS KIT VALEO at CES 2020. Valeo Innovations at the Epicenter of Transformations in Mobility. https://www.valeo.com/wp-content/uploads/2020/01/PK_Valeo_CES_2020_ENG-1.pdf.

[88] Li J., Liu X., Zhang W., Zhang M., Song J., Sebe N. (2020). Spatio-Temporal Attention Networks for Action Recognition and Detection. IEEE Trans. Multimed..

[89] Liu J., Zha Z.J., Chen X., Wang Z., Zhang Y. (2019). Dense 3D-convolutional neural network for person re-identification in videos. ACM Trans. Multimed. Comput. Commun. Appl. (TOMM).

[90] Trabelsi R., Varadarajan J., Zhang L., Jabri I., Pei Y., Smach F., Bouallegue A., Moulin P. (2019). Understanding the Dynamics of Social Interactions: A Multi-Modal Multi-View Approach. ACM Trans. Multimed. Comput. Commun. Appl. (TOMM).

[91] Russell B.C., Torralba A., Murphy K.P., Freeman W.T. (2008). LabelMe: A database and web-based tool for image annotation. Int. J. Comput. Vis..

[92] Wittenburg P., Brugman H., Russel A., Klassmann A., Sloetjes H. ELAN: A professional framework for multimodality research. Proceedings of the 5th International Conference on Language Resources and Evaluation (LREC 2006).

[93] Niitani Y., Akiba T., Kerola T., Ogawa T., Sano S., Suzuki S. Sampling Techniques for Large-Scale Object Detection From Sparsely Annotated Objects. Proceedings of the IEEE Conference on Computer Vision and Pattern Recognition (CVPR).

[94] Bolte J.A., Bar A., Lipinski D., Fingscheidt T. Towards corner case detection for autonomous driving. Proceedings of the 2019 IEEE Intelligent Vehicles Symposium (IV).

[95] Zhang C., Shang B., Wei P., Li L., Liu Y., Zheng N. Building Explainable AI Evaluation for Autonomous Perception. Proceedings of the IEEE Conference on Computer Vision and Pattern Recognition Workshops.

[96] Maddern W., Pascoe G., Linegar C., Newman P. (2017). 1 year, 1000 km: The Oxford RobotCar dataset. Int. J. Robot. Res..

[97] Chen C., Seff A., Kornhauser A., Xiao J. Deepdriving: Learning affordance for direct perception in autonomous driving. Proceedings of the IEEE International Conference on Computer Vision.

[98] Santana E., Hotz G. (2016). Learning a driving simulator. arXiv.

[99] Udacity (2017). Public Driving Dataset. https://www.udacity.com/self-driving-car.

[100] Madhavan V., Darrell T. (2017). The BDD-Nexar Collective: A Large-Scale, Crowsourced, Dataset of Driving Scenes. Master’s Thesis.

[101] Koschorrek P., Piccini T., Öberg P., Felsberg M., Nielsen L., Mester R. A multi-sensor traffic scene dataset with omnidirectional video. Ground Truth—What is a good dataset?. Proceedings of the IEEE Conference on Computer Vision and Pattern Recognition Workshops.

[102] Pandey G., McBride J.R., Eustice R.M. (2011). Ford campus vision and lidar data set. Int. J. Robot. Res..

[103] Brostow G.J., Fauqueur J., Cipolla R. (2009). Semantic object classes in video: A high-definition ground truth database. Pattern Recognit. Lett..

[104] Flohr F., Gavrila D. PedCut: An iterative framework for pedestrian segmentation combining shape models and multiple data cues. Proceedings of the 24th British Machine Vision Conference, BMVC.

[105] Dollar P., Wojek C., Schiele B., Perona P. Pedestrian detection: A benchmark. Proceedings of the 2009 IEEE Conference on Computer Vision and Pattern Recognition.

[106] Patil A., Malla S., Gang H., Chen Y.T. The H3D Dataset for Full-Surround 3D Multi-Object Detection and Tracking in Crowded Urban Scenes. Proceedings of the International Conference on Robotics and Automation.

[107] Yao Y., Xu M., Choi C., Crandall D.J., Atkins E.M., Dariush B. Egocentric Vision-based Future Vehicle Localization for Intelligent Driving Assistance Systems. Proceedings of the International Conference on Robotics and Automation.

[108] Xu M., Gao M., Chen Y.T., Davis L.S., Crandall D.J. Temporal recurrent networks for online action detection. Proceedings of the IEEE International Conference on Computer Vision.

[109] Chang W.C., Cho C.W. (2009). Online boosting for vehicle detection. IEEE Trans. Syst. Man Cybern. Part B (Cybern.).

[110] Sivaraman S., Morris B., Trivedi M. Learning multi-lane trajectories using vehicle-based vision. Proceedings of the 2011 IEEE International Conference on Computer Vision Workshops (ICCV Workshops).

[111] Cherng S., Fang C.Y., Chen C.P., Chen S.W. (2009). Critical motion detection of nearby moving vehicles in a vision-based driver-assistance system. IEEE Trans. Intell. Transp. Syst..

[112] Alonso J.D., Vidal E.R., Rotter A., Muhlenberg M. (2008). Lane-change decision aid system based on motion-driven vehicle tracking. IEEE Trans. Veh. Technol..

[113] Zhu Y., Comaniciu D., Pellkofer M., Koehler T. (2006). Reliable detection of overtaking vehicles using robust information fusion. IEEE Trans. Intell. Transp. Syst..

[114] Wang J., Bebis G., Miller R. Overtaking vehicle detection using dynamic and quasi-static background modeling. Proceedings of the 2005 IEEE Computer Society Conference on Computer Vision and Pattern Recognition (CVPR’05)-Workshops.

[115] Barth A., Franke U. (2009). Estimating the driving state of oncoming vehicles from a moving platform using stereo vision. IEEE Trans. Intell. Transp. Syst..

[116] Rabe C., Franke U., Gehrig S. Fast detection of moving objects in complex scenarios. Proceedings of the 2007 IEEE Intelligent Vehicles Symposium.

[117] Barth A., Franke U. Tracking oncoming and turning vehicles at intersections. Proceedings of the 13th International IEEE Conference on Intelligent Transportation Systems.

[118] Hermes C., Einhaus J., Hahn M., Wöhler C., Kummert F. Vehicle tracking and motion prediction in complex urban scenarios. Proceedings of the 2010 IEEE Intelligent Vehicles Symposium.

[119] Taha A., Chen Y.T., Yang X., Misu T., Davis L. (2019). Exploring Uncertainty in Conditional Multi-Modal Retrieval Systems. arXiv.

[120] Taha A., Meshry M., Yang X., Chen Y.T., Davis L. (2018). Two stream self-supervised learning for action recognition. arXiv.

[121] Cui Z., Heng L., Yeo Y.C., Geiger A., Pollefeys M., Sattler T. Real-time dense mapping for self-driving vehicles using fisheye cameras. Proceedings of the 2019 International Conference on Robotics and Automation (ICRA).

[122] Wiest J., Höffken M., Kreßel U., Dietmayer K. Probabilistic trajectory prediction with Gaussian mixture models. Proceedings of the 2012 IEEE Intelligent Vehicles Symposium.

[123] Qiu Y., Misu T., Busso C. Driving Anomaly Detection with Conditional Generative Adversarial Network using Physiological and CAN-Bus Data. Proceedings of the 2019 International Conference on Multimodal Interaction.

[124] Tawari A., Mallela P., Martin S. Learning to Attend to Salient Targets in Driving Videos Using Fully Convolutional RNN. Proceedings of the 2018 21st International Conference on Intelligent Transportation Systems (ITSC).

[125] Taha A., Chen Y.T., Misu T., Shrivastava A., Davis L. (2019). Unsupervised Data Uncertainty Learning in Visual Retrieval Systems. arXiv.

[126] Zhao B., Luo G. A New Causal Direction Reasoning Method for Decision Making on Noisy Data. Proceedings of the 2019 IEEE Intelligent Vehicles Symposium (IV).

[127] Dua I., Nambi A.U., Jawahar C., Padmanabhan V. AutoRate: How attentive is the driver?. Proceedings of the 2019 14th IEEE International Conference on Automatic Face & Gesture Recognition (FG 2019).

[128] Fontana V., Singh G., Akrigg S., Di Maio M., Saha S., Cuzzolin F. (2018). Action Detection from a Robot-Car Perspective. arXiv.

[129] Misu T., Chen Y.T. Toward Reasoning of Driving Behavior. Proceedings of the 2018 21st International Conference on Intelligent Transportation Systems (ITSC), 14th IEEE International Conference on Automatic Face & Gesture Recognition (FG 2019).

[130] Nica A.N., Trascau M., Rotaru A.A., Andreescu C., Sorici A., Florea A.M., Bacue V. Collecting and Processing a Self-Driving Dataset in the UPB Campus. Proceedings of the 2019 22nd International Conference on Control Systems and Computer Science (CSCS).

[131] Martin M., Roitberg A., Haurilet M., Horne M., Reiß S., Voit M., Stiefelhagen R. Drive&Act: A Multi-modal Dataset for Fine-grained Driver Behavior Recognition in Autonomous Vehicles. Proceedings of the IEEE International Conference on Computer Vision.

[132] Li C., Xu M., Du X., Wang Z. Bridge the Gap Between VQA and Human Behavior on Omnidirectional Video: A Large-Scale Dataset and a Deep Learning Model. Proceedings of the 26th ACM International Conference on Multimedia (MM’18).

[133] Zhan W., Sun L., Wang D., Shi H., Clausse A., Naumann M., Kummerle J., Konigshof H., Stiller C., de La Fortelle A. (2019). INTERACTION Dataset: An INTERnational, Adversarial and Cooperative moTION Dataset in Interactive Driving Scenarios with Semantic Maps. arXiv.

[134] He K., Zhang X., Ren S., Sun J. (2016). Identity mappings in deep residual networks. Proceedings of the European Conference on Computer Vision.

